# Supplementary figures and images for: Dissecting the Genetic Basis of a Complex cis-Regulatory Adaptation
Source: PLoS Genet. 2015 Dec 29;11(12):e1005751. doi: 10.1371/journal.pgen.1005751 (PMC4694769; doi:10.1371/journal.pgen.1005751)

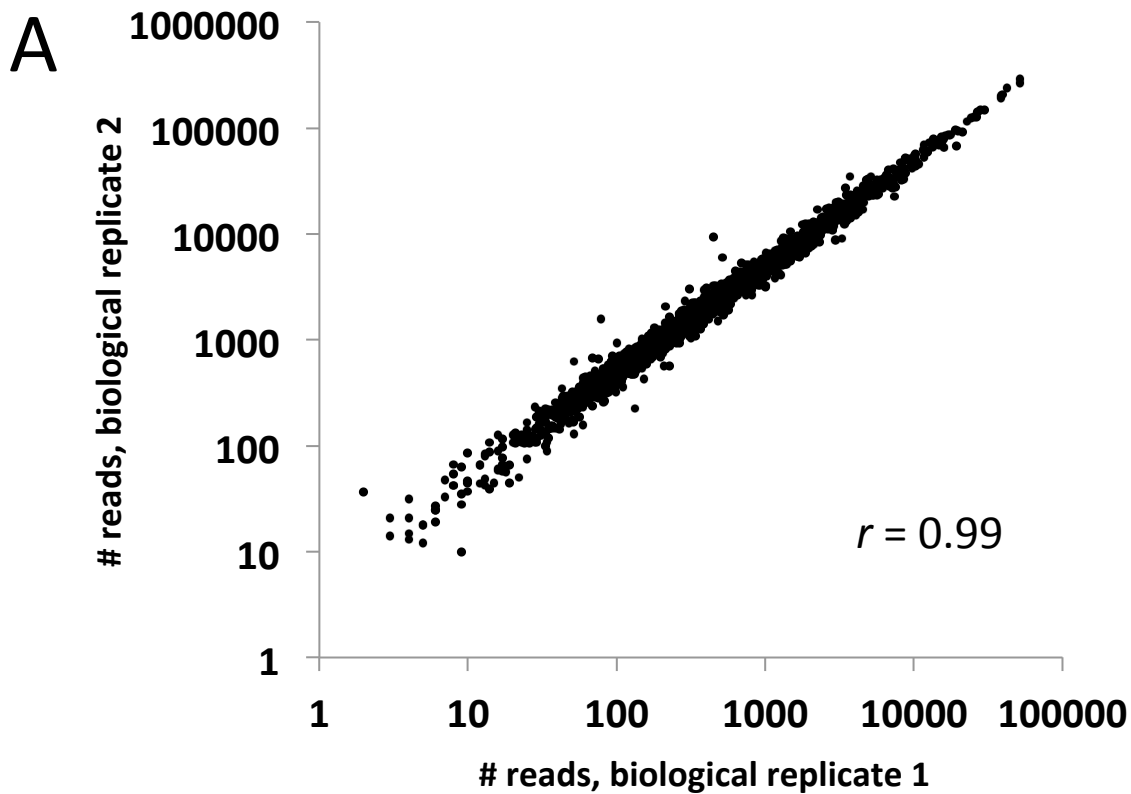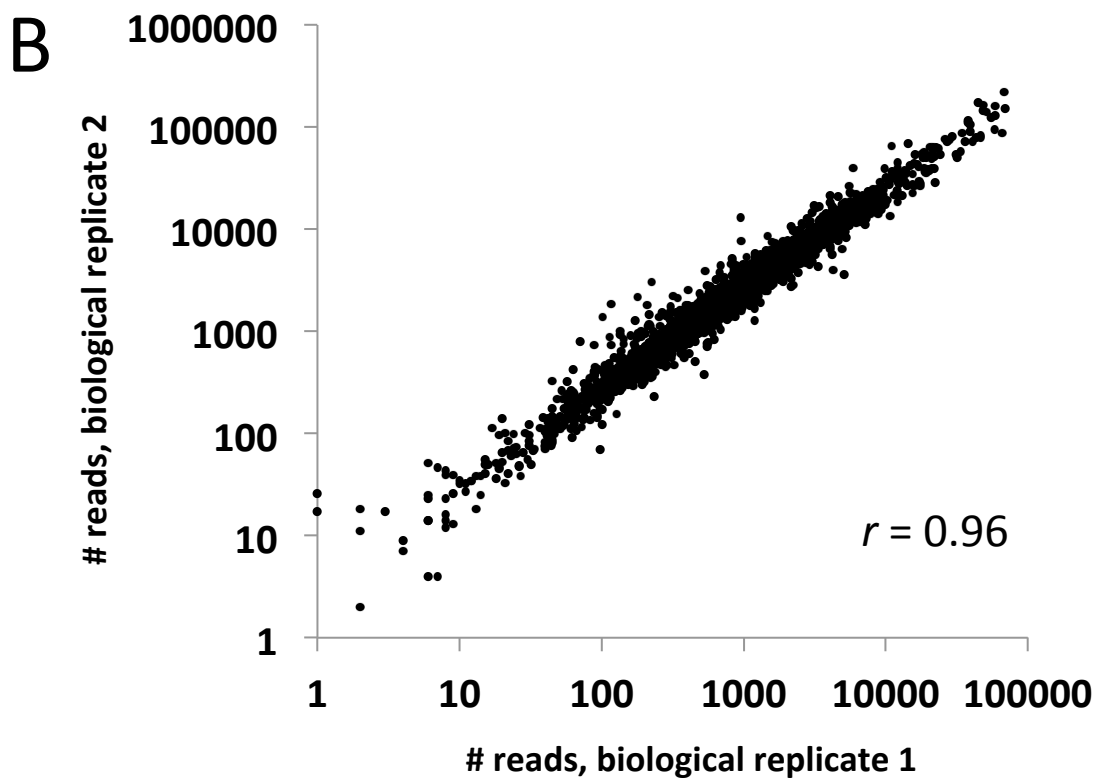

Supplement: S1 Fig — A. Reads per gene are shown for RNA-seq replicates of the Sc/Sp hybrid grown in YPD + DMSO. B. Reads per gene are shown for RNA-seq replicates of the Sc/Sp hybrid grown in YPD + 600 ppm citrinin (dissolved in DMSO). (PDF) [file pgen.1005751.s001.pdf]

**A**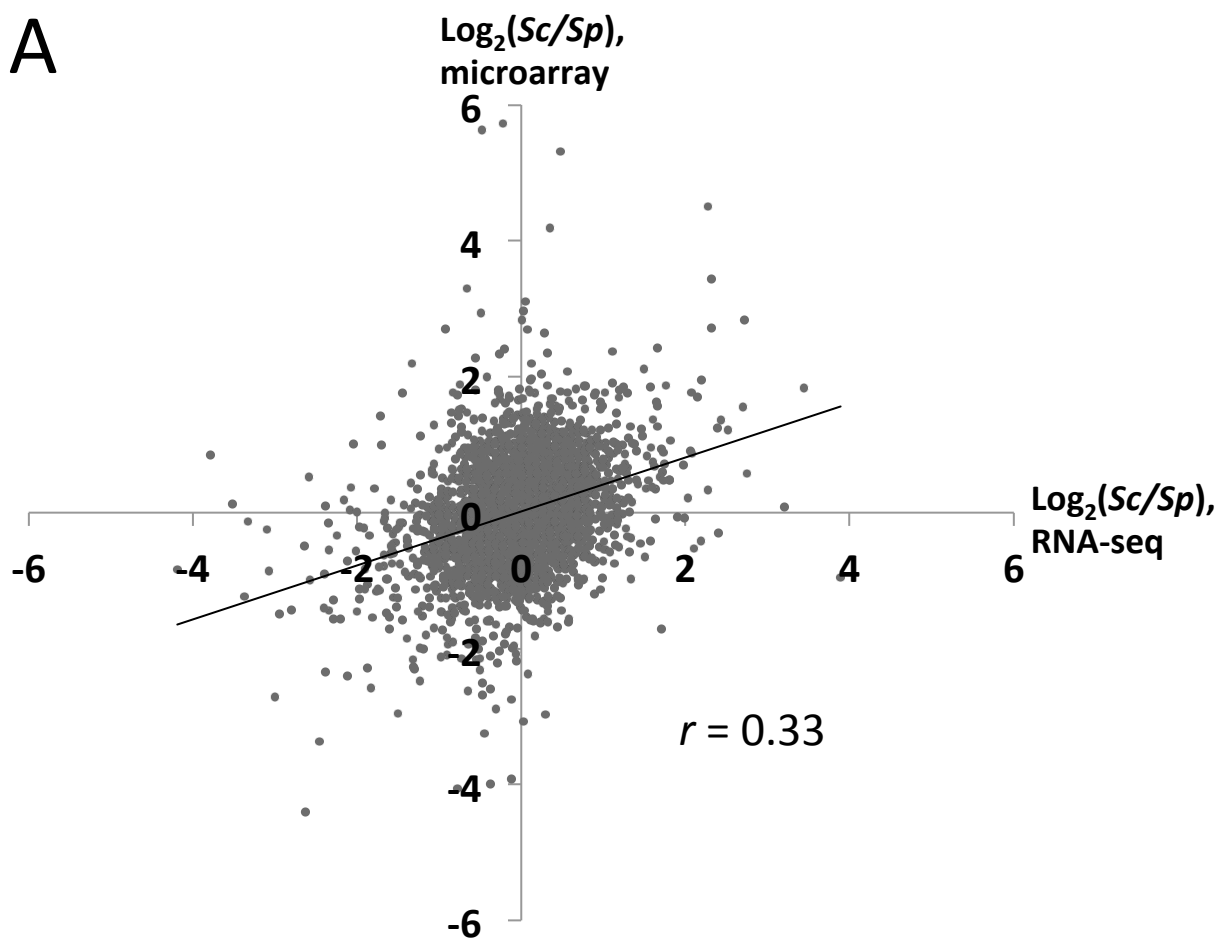**B**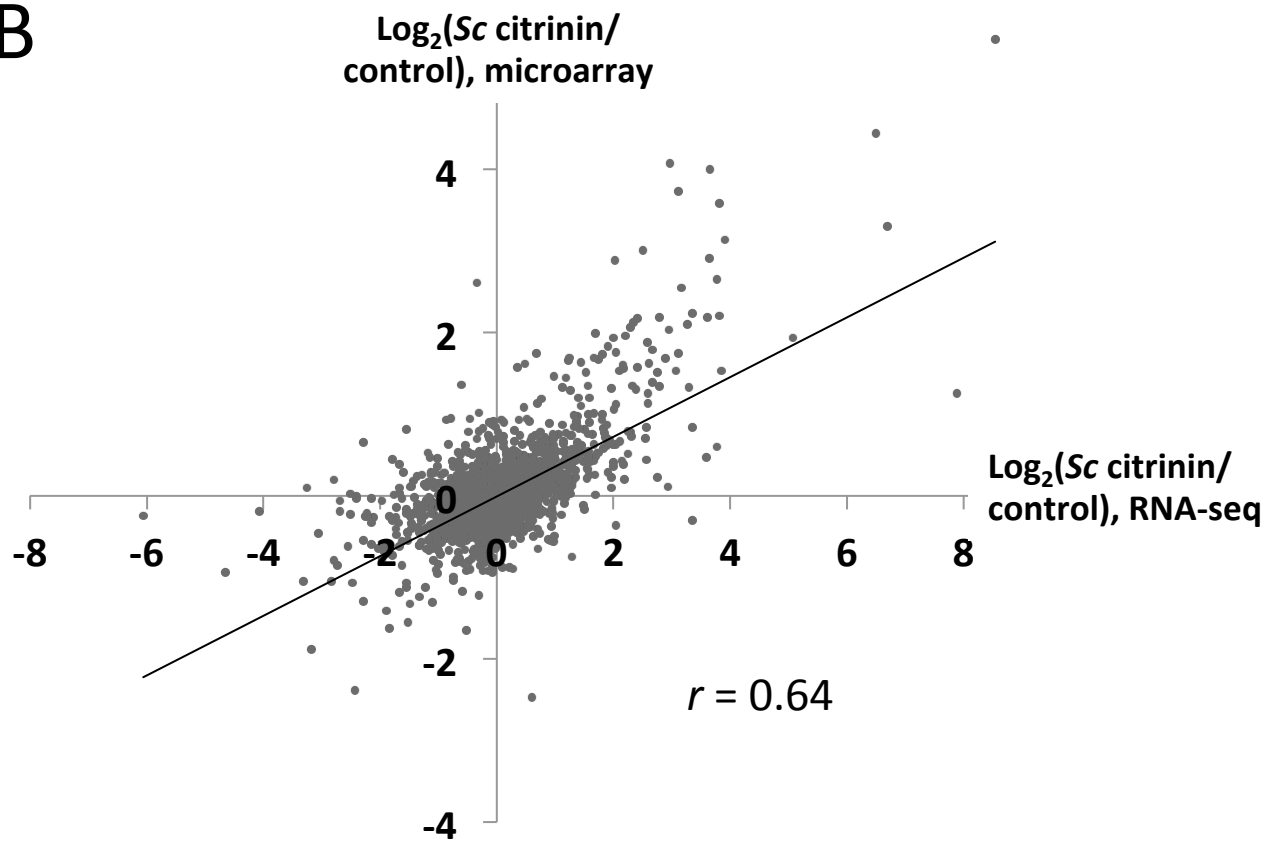

Supplement: S2 Fig — A. ASE ratios for the Sc/Sp hybrid in microarray data in YPD [16] compared to our RNA-seq data in YPD+DMSO. B. Fold-changes for Sc’s response to 300 ppm citrinin from oligonucleotide microarray data [15], compared to the Sc allele’s response (within the Sc/Sp hybrid) to 600 ppm citrinin in our RNA-seq data. (PDF) [file pgen.1005751.s002.pdf]
